# Upper-Body Muscular Endurance Training Improves Performance Following 50 min of Double Poling in Well-Trained Cross-Country Skiers

**DOI:** 10.3389/fphys.2017.00690

**Published:** 2017-09-22

**Authors:** Jørgen Børve, Steffen N. Jevne, Bjarne Rud, Thomas Losnegard

**Affiliations:** Department of Physical Performance, Norwegian School of Sport Sciences Oslo, Norway

**Keywords:** cross-country skiing, high-intensity training, maximal oxygen uptake, O_2_-cost, running, training intensity

## Abstract

This study investigated the effect of muscular endurance training on O_2_-cost and performance in double poling (DP) on a rollerski treadmill. Twenty-two well-trained cross-country skiers (31 ± 4 years, 77 ± 9 kg, 181 ± 8 cm, VO_2max_ running: 64 ± 5 mL·kg^−1^·min^−1^) were counter-balanced to either a combined muscular endurance and running interval training group [MET; *n* = 11 (♂ = 9, ♀ = 2)], or an endurance running interval training group [ET; *n* = 11 (♂ = 9, ♀ = 2)]. Both groups continued their normal low-and moderate intensity training, but replaced 2 weekly high intensity-training sessions with two project-specific sessions for 6 weeks. In these sessions, MET combined upper-body muscular endurance training (4 × 30 repetitions, 90 s rest between sets) and running intervals (3 × 4 or 2 × 6 min, 3 min rest), while ET performed running intervals only (6 × 4 or 4 × 6 min, 3 min rest). The DP test-protocol consisted of 50 min submaximal poling for O_2_-cost measurement, followed by a self-paced 1,000-m performance test. In addition, subjects performed a VO_2max_ test in running. MET increased muscular endurance (*P* < 0.05) and 1RM in simulated DP (*P* < 0.01) more than ET. Further, MET reduced the 1,000-m time and O_2_-cost compared to baseline values (*P* < 0.05), and tended to improve the 1,000-m time more than ET (*P* = 0.06). There were no changes in VO_2max_ running or VO_2peak_ DP in either MET or ET. In conclusion, 6 weeks of muscular endurance training increased both muscular endurance and 1RM in simulated DP. Further, specific upper-body muscular endurance training improved DP performance and thus, seems as a promising training model to optimize performance in well-trained cross-country skiers.

## Introduction

In cross-country (XC) skiing, the classic style double poling (DP) technique has been considerably developed over the last decade and is today the main technique used in races. The DP technique is characterized by a symmetrical DP action, which transfers propulsive forces solely through the poles. This emphasizes the importance of well-developed upper-body power in employing DP successfully throughout an entire race (Stöggl et al., 2007; Losnegard et al., 2011). Consequently, specific upper-body training, both in research and practical situations, has gained interest as a training model for improving such abilities (Nilsson et al., 2004; Terzis et al., 2006; Losnegard et al., 2011; Skattebo et al., 2015).

The introduction of new competition formats such as sprint and mass starts has also increased the importance of a high work-intensity during the closing part of races, which makes it vital to conserve power for the final sprint. This requirement could potentially be met by working at a lower relative intensity during the submaximal part of the competition, resulting in less fatigued muscles during the final sprint (Bassett and Howley, 2000). However, limited data are available on which type of training is most efficient in improving such abilities in XC skiing generally and DP more specifically.

In previous research, the main training model for developing upper-body power to improve DP performance has been heavy strength training (≤12 repetition maximum). However, these studies have yielded varying results, displaying both large (Hoff et al., 1999, 2002; Østeras et al., 2002) and trivial effects (Losnegard et al., 2011; Skattebo et al., 2015). Furthermore, the metabolic response during DP seems to be different for the arms and legs, indicating that intense upper-body endurance training may increase the arms' ability to extract oxygen and thus enhance DP performance (Rud et al., 2014).

The effect of muscular endurance training (20–100 reps/set, Campos et al., 2002) on endurance performance has been investigated in various sports including running (Mikkola et al., 2011; Sedano et al., 2013) and rowing (Ebben et al., 2004; Gallagher et al., 2010). Such training seem to target different muscular and neurological adaptations compared to heavy strength training; e.g., mitochondria and capillary density, muscle fiber composition and cross-sectional area (Campos et al., 2002). Specifically for XC skiing, Nilsson et al. (2004) showed that 20-s or 180-s interval training in a DP ergometer increased both 30-s and 6-min power output in well-trained XC skiers, while Vandbakk et al. (2017) demonstrated increased time to exhaustion after 8 weeks of 30-s DP intervals. This indicates that upper-body power training might have the potential to increase performance in XC skiing, although data on the effect on finishing abilities is limited.

In terms of training, three variables (intensity, duration, and frequency) together set the training load with the explicit goal of maximizing performance (Seiler, 2010). When investigating the effect of a specific training program for recreational athletes, often with limited time to execute training, it seems important to keep the three variables similar before and during interventions to determine the effect of the intervention itself. Otherwise, it could be unclear whether improved performance is a result of adaptations related to the added training, or changed training load in general. The aim of the present study was, therefore, to investigate the effect of replacing parts of high intensity interval training with muscular endurance training for 6 weeks on performance after completing 50 min of submaximal DP. The main hypotheses of the present study were; Upper body muscular endurance training would (I) improve performance following 50-min of double poling, and (II) reduce O_2_-cost during submaximal double poling.

## Materials and methods

### Subjects

During the intervention, two men withdrew from the project due to unrelated reasons. In total, 22 well-trained XC skiers (18 males and 4 females) completed the study with the required number of completed project-specific training sessions (minimum adherence 85%; MET = 94 ± 8% adherence; ET = 90 ± 5% adherence). Inclusion criteria were completing the long-distance XC ski race *Birkebeinerrennet* the previous year with a finishing time <4 h and < 3 h 30 min for females and males respectively. None of the subjects performed specific upper-body muscular endurance training systematically prior to the start of the study, but all performed weekly aerobic high-intensive interval training. After completing the pre-test, participants were counter-balanced to either a muscular endurance training group (MET: 30 ± 4 years, 76 ± 8 kg, 180 ± 7 cm) or an endurance training group (ET: 32 ± 4 years, 78 ± 11 kg, 182 ± 10 cm) based on the following pre-test characteristics: DP 1,000-m time, VO_2max_ running, 1 repetition maximum (1RM) and gender. All skiers gave their written informed consent before participating. The study was conducted according to the Declaration of Helsinki and Norwegian law.

### Design

The investigation was conducted during the pre-competition period for XC skiers (August–November), and contained a 6-week intervention period, enclosed by a pre- and post-test (Table 1). The week before pre-tests, subjects performed one familiarization session on the rollerski treadmill which consisted of three different submaximal speeds, followed by a 1,000-m performance test (see *Prolonged double poling protocol*). Then, a familiarization session with the muscular endurance exercise in standing DP was performed, using the cable-pulley and consisted of a warm-up and 1RM test (2–3 attempts). Pre- and post-tests were conducted in the week immediately before and after the training period and included 2 test days for each participant, separated by at least 48 h. Subjects were not allowed to perform any strength training the day before testing, and only a maximum of 90 min endurance training at low intensity. Day 1 included a running VO_2max_ test followed by an upper-body 1RM and a muscular endurance test in a cable-pulling apparatus. Day 2 included a submaximal and maximal test in DP on a rollerski treadmill. During the intervention period, both groups replaced two of their weekly high-intensity interval training (HIT) sessions with a specific protocol based on their respective group. For MET, these sessions consisted of both running intervals and upper-body muscular endurance training, while for ET it involved two sessions with running intervals only. During the other weekly training sessions, the subjects were encouraged to maintain their normal training routines.

**Table 1:** The test-battery and time-line of the study.

Familiarization	x								
Test day 1:									
VO_2max_running test		x							x
1RM- and muscular endurance test		x							x
Test day 2:									
Double poling protocol		x							x
		Pre-test	Intervention						Post-test
		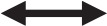	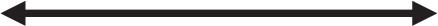						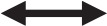
Week:	−2	−1	1	2	3	4	5	6	7

*VO_2max_, maximal oxygen uptake; RM, repetition maximum*.

### Running VO_2max_ (day 1)

Following a 20-min warm up (60–85% of HR_peak_), subjects performed a VO_2max_ running test at a 10.5% incline with stepwise increments of 1 km-h^−1^ every minute until volitional exhaustion. Starting speed was set individually based on subjects' race history (average: 9.4 ± 0.7 km-h^−1^), but was similar for each subject at pre- and post-test. All tests lasted 4.5–7.5 min, and the highest VO_2_ over a 60 s period was considered as VO_2max_.

### Maximal strength and muscular endurance (day 1)

**Figure 1:**
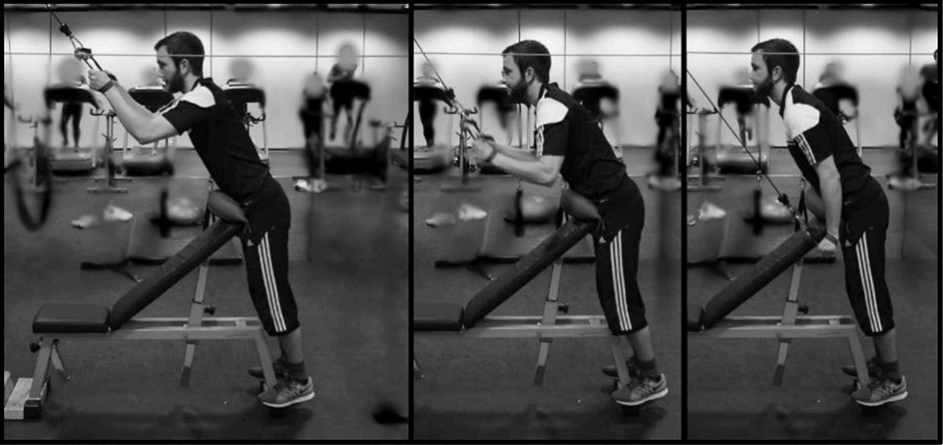
The standing double poling exercise used during testing and training. The figure illustrate the poling phase of the cycle. The participant gave his consent to publish the picture.

Fifteen minutes after the running VO_2max_ test, the subjects performed an exercise-specific warm-up in the standing DP, consisting of four sets (15 reps at 40%, 10 reps at 55%, 5 reps at 75%, 3 reps at 85% of 1RM as estimated during familiarization). In the 1RM test, the load was set to 95% of estimated 1RM (at post-test, 95% of pre-test 1RM was used), and increased 2–5% after each successful attempt (3 min break) until the subject failed on two consecutive lifts. The heaviest successful attempt was considered 1RM. After a 5 min break, the muscular endurance test was conducted at 55% of individual 1RM. This relative workload was set based on pilot testing and previous studies, with the aim of completing more than 20, but less than 100 repetitions (Stone and Coulter, 1994; Campos et al., 2002). The test was performed using a constant DP motion (cycle time; pre 1.4 ± 0.2 s; post 1.4 ± 0.2 s, based on video recording) until exhaustion. All testing and training with the cable-pulley was done with a customized handlebar to simulate a DP grip (Figure 1).

### Prolonged double poling protocol (day 2)

**Figure 2:**
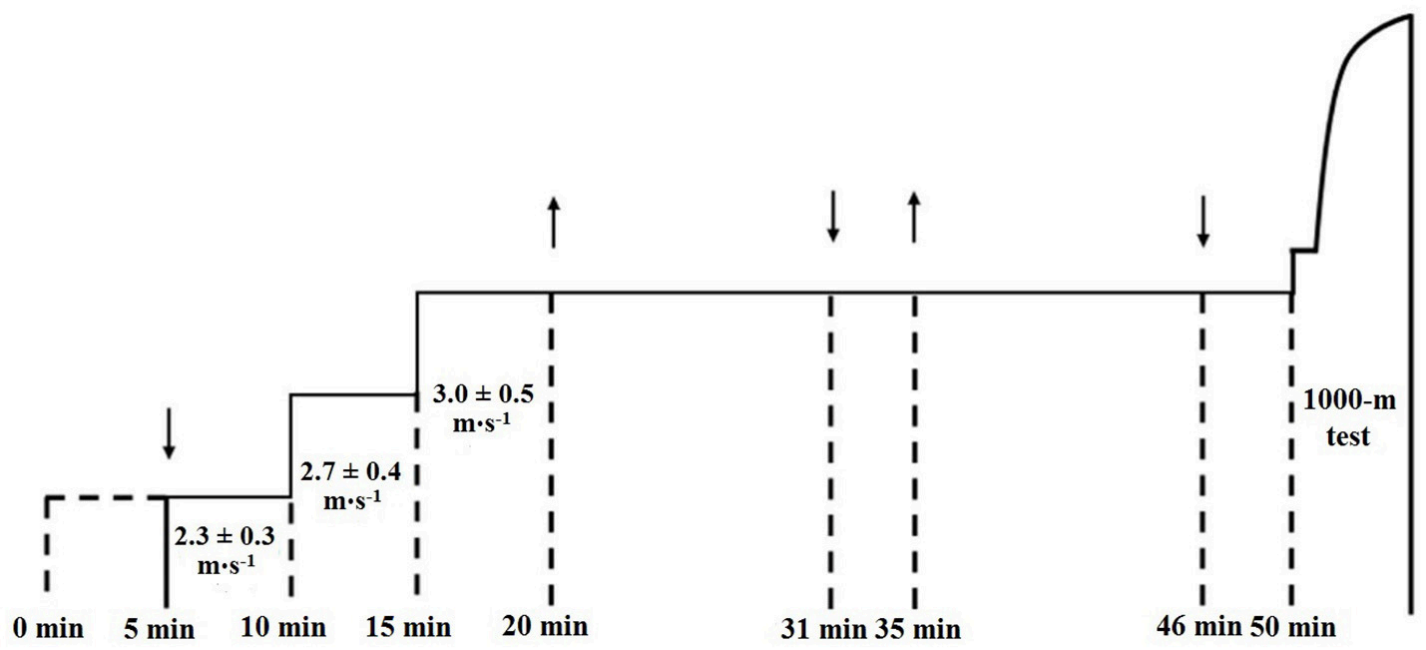
Schematic illustration of prolonged double poling protocol. Steady-state VO_2_ was measured from 5 to 20, 31 to 35, and 46 to 50 min followed by a self-paced 1,000-m maximal test. ↓ = start O_2_ measurements. ↑ = end O_2_measurements. All tests conducted at 2.5° incline and the speed shown is the average from both groups. Speed from 0 to 5 min was identical to 5 to 10 min.

The protocol was initiated by a 5 min trial identical to the first submaximal speed to ensure a steady state oxygen uptake at the first submaximal load. Further, subjects performed three different submaximal speeds (50 min total), followed directly by a 1,000-m test (Figure 2). All DP tests were conducted at 2.5°. Submaximal speeds were based on the linear relationship between workload and O_2_-cost, with the aim of matching speed to 75% of VO_2peak_ DP from 15 to 50 min and adjusted with the RPE (rate of perceived exertion, Borg, 1982) with the aim of not exceeding 16 on the RPE-scale before the 1,000-m test. Thus, speeds were individually set, but were identical for each participant in the pre- and post-test. The average speed for all participants at submaximal speeds one, two and three was 2.3 ± 0.3, 2.7 ± 0.4 and 3.0 ± 0.5 m·s^−1^, respectively. The O_2_-cost was calculated from 8-10, 13-15, 18-20, 33-35 and 48-50 min during the prolonged submaximal test. Heart rate (HR) was registered after 10, 15, 20, 35, and 50 min and RPE after 20, 35, and 45 min. The 1,000-m test was a modified protocol from Losnegard et al. (2013). In brief, a fixed individual speed, identical at pre- and post-test, was set for the initial 200 m to avoid over-pacing (avg. 3.41 ± 0.5 m·s^−1^). Thereafter, the subjects were free to adjust their speed based on their position on the treadmill (0.25 m·s^−1^ increase or decrease). VO_2_ and HR were measured continuously, and the highest VO_2_ and HR (avg. 30 s) were considered peak values. RPE was reported immediately after finishing the test. During the prolonged exercise, participants were asked to drink 3 dl of water.

### Apparatus

Oxygen consumption was measured with an automatic ergospirometry system (Oxycon Pro, Jaeger Instrument, Hoechberg, Germany), as evaluated by Foss and Hallen (2005). The gas analysers and the flow turbine (Tripel V; Erick Jeager GmbH, Hoechberg, Germany) were calibrated before each test according to the instruction manual, as described previously (Losnegard et al., 2011). The same gas analyser was used during the running VO_2max_ test and DP protocol. Heart rate was recorded using a Polar V800 (Polar Electro Oy, Kempele, Finland). A rollerski treadmill with belt dimension 3 × 4.5 m (Rodby, Södertelje, Sweden), was used during the running VO_2max_ test and the prolonged DP test. Two different pairs of rollerskis (Swenor Fiberglass, Trøsken, Norway), with front wheel type 2 and rear wheel type 3, were used depending on which ski binding system the skiers used (SNS, Salomon, Annecy, France or NNN, Rottefella, Klokkarstua, Norway). The rollerski friction coefficient was 0.026 before, during and after the project, and tested as described in Hoffman et al. (1990). All participants used Swix Triac 1 poles (Swix, Lillehammer, Norway), with customized tips for rollerski treadmills (self-selected pole length for all tests; 153.3 ± 7.7 cm, corresponding to 82 ± 3% of body height). A cable-pulley machine (Technogym Cable Jungle, Gambettola, Italy), with a 45 cm-wide custom-made handlebar (Losnegard et al., 2011) was used during training and tests in the standing DP. Cycle times during submaximal DP (after 21 and 41 min) and the muscular endurance test were recorded (Sony DCR-TRV900E; Sony, Tokyo, Japan).

### Training

A description of the specific training sessions is provided in Table 2. The training sessions lasted approx. 50 min, including a 10 min warm-up. Running intervals lasted 4 or 6 min with 3 min rest periods in-between. During the muscular endurance training, there was a passive rest of 90 s between sets. Speed during running intervals was based on individual HR_max_ measured during the VO_2max_ running test, and adjusted to match the intended HR-zone within 90 s of trial-start (88–92% of HR_max_) (Helgerud et al., 2007). The intended intensity has previously been shown to be advantageous in increasing the maximal oxygen uptake in well-trained athletes (Seiler et al., 2013). All subjects where individually supervised during the 1st, 5th, and 9th training session to ensure proper intensity during intervals. Additionally, MET was observed in simulated DP to ensure proper technique and the load was adjusted according to Table 2. All training from the 4 weeks prior to the beginning of the intervention until the end of the intervention period were reported. The endurance training intensity was divided into three HR zones: low-intensity (LIT; 60–81% of peak HR_max_), moderate-intensity (MIT; 82–87%) and high-intensity training (HIT 88–100%), based on intensity zones developed by the Norwegian Olympic Federation.

**Table 2:** Training programs for the 2 weekly project sessions in MET (Combined endurance training and muscular endurance training) and ET (endurance training).

**Week**	**MET**					**ET**	
	**Running interval**		**Muscular endurance**			**Running interval**	
	**Duration (sets × min)**	**Intensity (% HR_peak_)**	**Sets**	**Rep**	**Workload (% 1 RM)**	**Duration (sets × min)**	**Intensity (% of HR_peak_)**
1–2	3 × 4/2 × 6	88–92	4	30	42.5	6 × 4/4 × 6	88–92
3–4	3 × 4/2 × 6	88–92	4	30	45	6 × 4/4 × 6	88–92
5–6	3 × 4/2 × 6	88–92	4	30	47.5	6 × 4/4 × 6	88–92

*During running intervals, a 3 min rest was applied*.

*HR_peak_, peak heart rate; RM, repetition maximum*.

### Statistical analysis

All data are presented as mean ± standard deviation (SD). Baseline differences between groups were tested with unpaired Student's *t*-tests. Within-group and between-group changes were tested with paired Student's *t*-tests and two-way repeated-measures analysis of variance (ANOVA), respectively. All statistical analysis was performed using Excel 2003 (Microsoft Corporation, Redmond, Washington, USA) and IBM SPSS Statistics 20 (International Business Machines, New York, USA). The level of confidence was set to 90% and *P* ≤ 0.05 were considered statistically significant, while 0.05 < *P* ≤ 0.1 was considered a tendency toward statistical significance.

## Results

### Training

There was a tendency toward differences between groups in total training volume prior to the training intervention (*P* = 0.10), mainly due to a higher volume of LIT in MET compared to ET (Table 3). There were no differences in the intensity-zone distribution between pre-intervention and intervention in MET. In ET, there was a small, but significant (0.1 h) increase in total training volume of HIT per week from pre-intervention to intervention.

**Table 3:** Training distribution (hours per week) in the 4 weeks prior to pre-test (Pre-intervention) and during the 6-week intervention period for MET (*n* = 11) and ET (*n* = 11).

**Variable**	**MET**		**ET**	
	**Pre-intervention**	**Intervention**	**Pre-intervention**	**Intervention**
LIT (60–81% of HR_peak_)	6.4 ± 3.1	5.6 ± 2.9	4.3 ± 2.2	4.1 ± 2.3
MIT (82–87% of HR_peak_)	1.2 ± 0.6	1.2 ± 0.7	1.0 ± 0.5	1.0 ± 0.6
HIT (88–100% of HR_peak_)	0.5 ± 0.2	0.6 ± 0.3	0.6 ± 0.3	0.7 ± 0.2^*^
Total endurance training	8.1 ± 3.6	7.5 ± 3.3	5.9 ± 2.1	5.8 ± 2.0
Muscular endurance training	–	0.3 ± 0.1	–	–
Strength training	0.5 ± 0.7	0.5 ± 0.5	0.3 ± 0.4	0.4 ± 0.5
Total training	8.6 ± 4.6	8.3 ± 4.2	6.2 ± 3.2	6.2 ± 3.1
Double poling	3.3 ± 2.7	2.4 ± 2.4	1.5 ± 1.1	1.2 ± 0.8

LIT, low-intensity training; MIT, moderate intensity training; HIT, high-intensity training; MET, combined muscular endurance and endurance training group; ET, endurance training group.

* *Significant in-group difference from pre-intervention to intervention (P < 0.05). Double poling is performed during rollerski*.

### Muscular endurance and maximal strength

**Figure 3:**
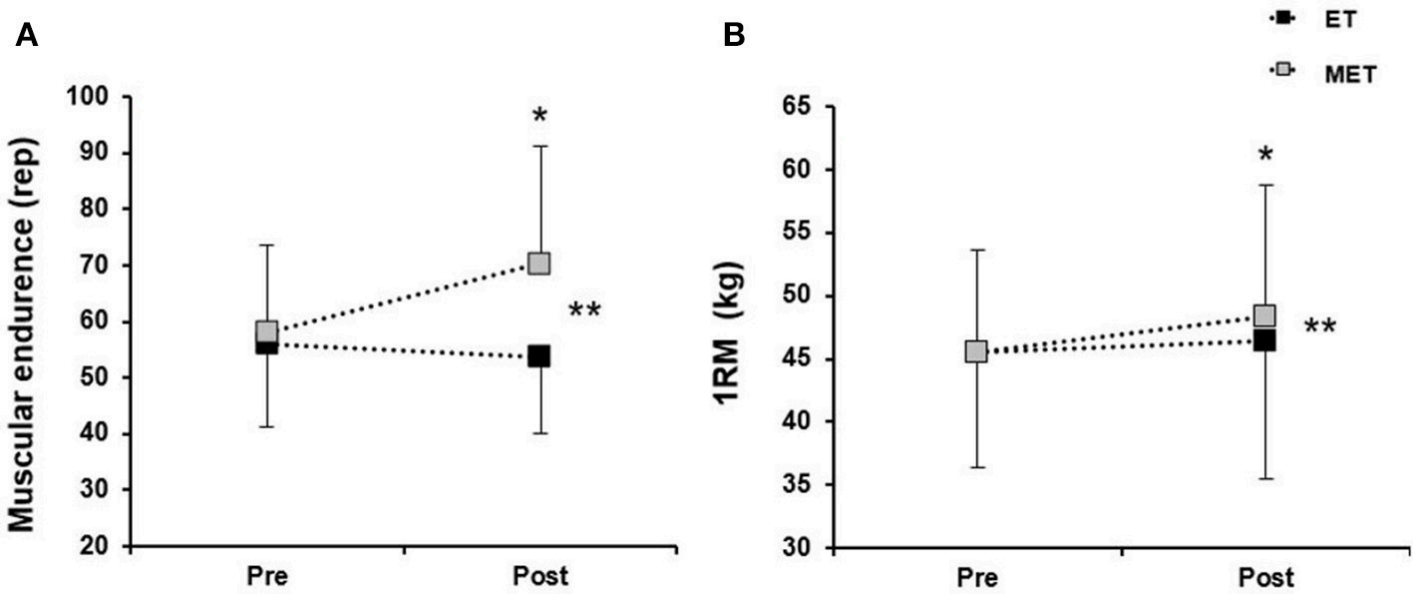
Muscular endurance **(A)** and 1RM **(B)** in the standing double poling exercise before (pre) and after (post) 6 weeks of combined endurance- and muscular endurance training (MET) or endurance training (ET). Data are expressed as group mean ± standard deviation. ^*^Significant change from pre- to post-test (*P* < 0.05). ^**^Significant differences from pre- to post-test between MET and ET (*P* < 0.05).

MET improved muscular endurance by 21 ± 8% (mean ± 90% confidence interval) while no changes occurred in ET (−1 ± 11%), resulting in a 22 ± 8% difference between groups (Figure 3A). Individual differences from pre- to post-test are shown in **Figure 7B**. 1RM increased significantly in MET (6 ± 2%) and tended to increase in ET (2 ± 3%; *P* = 0.05), with a significant group difference of 4 ± 4% (Figure 3B).

### 1,000-m time

**Figure 4:**
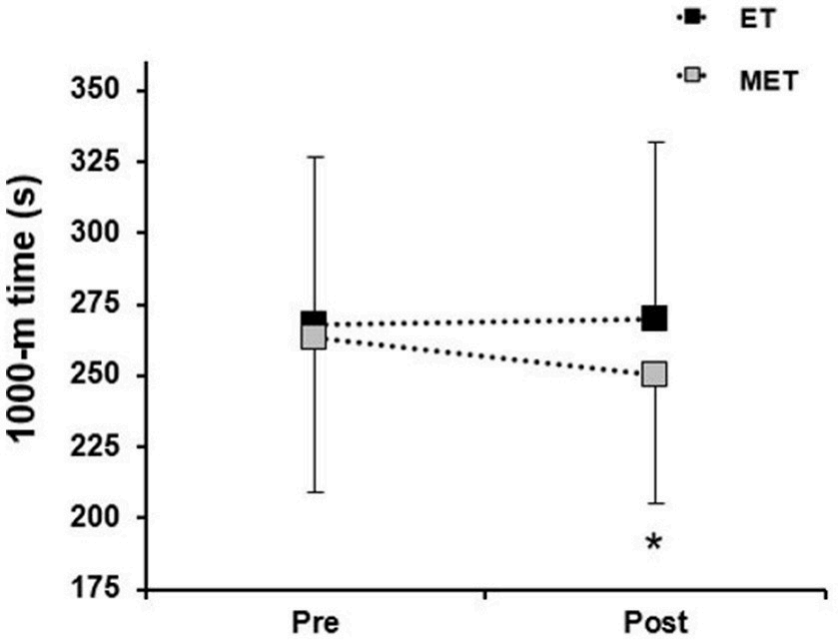
1,000-m time before (pre) and after (post) 6 weeks of combined endurance training and muscular endurance training (MET) and endurance training (ET). Data are expressed as group mean ± standard deviation. ^*^Tendency to change from pre- to post-test (*P* = 0.06).

**Figure 5:**
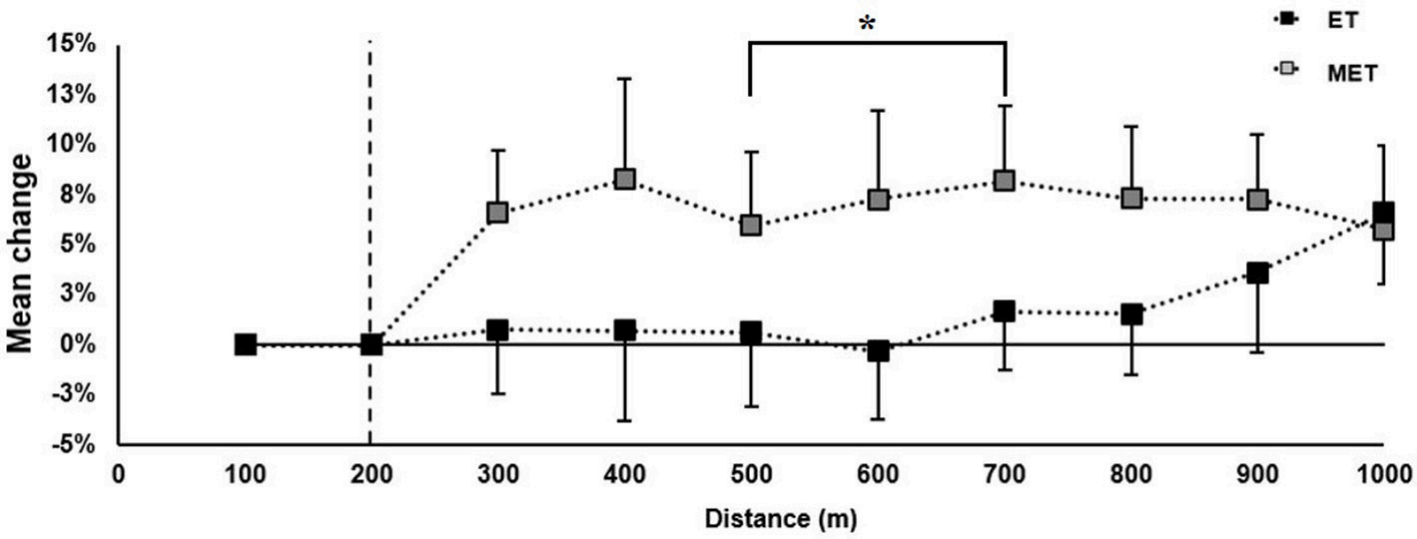
Relative change in speed during the 1,000-m time trial from pre- (black horizontal line at 0%) to post-test. Data are expressed as group mean ± standard deviation. The speed was set the first 200 m. All tests conducted at 2.5° incline. ^*^Significant differences from pre- to post-test between combined endurance training and muscular endurance training (MET) and endurance training (ET) (*P* < 0.05).

The mean 1,000-m time decreased in MET (−4 ± 2%), but was unchanged in ET (−1 ± 2%, Figure 4), which resulted in a between-group difference of 4 ± 2% (*P* = 0.06). Individual differences from pre- to post-test are shown in **Figure 7A**. From pre- to post-test, MET increased speed more than ET between 500 and 700-m (7 ± 1%), and there was a tendency to difference between groups at intervals 200–400-m (5 ± 1%, *P* = 0.08) and 700–800-m (5 ± 1%, *P* = 0.08) (Figure 5).

### Physiological responses during 1,000-m time and VO_2max_ running

VO_2max_ running and VO_2peak_ DP was unchanged from pre- to post-test in both groups (Table 4). Individual changes in VO_2peak_ DP are shown in **Figure 7C**. The percent differences between VO_2peak_ DP and VO_2max_ running were 89 ± 3% in MET and 89 ± 2% in ET at both pre- and post-test. There was a tendency toward increase in HR_peak_ DP in MET (2 ± 1%; *P* = 0.06), while ET remained unchanged (−1 ± 1%), resulting in a significant difference of 3 ± 1% between groups. Rating of perceived exertion (RPE) after the 1,000-m test was unchanged from pre- to post-test in both groups.

**Table 4:** Physiological response during the 1,000-m time test (MET: *n* = 11; ET: *n* = 10) and running VO_2max_ test (MET: *n* = 11; ET: *n* = 11). Data are mean ± standard deviation.

**Variable**	**MET**		**ET**	
	**Pre**	**Post**	**Pre**	**Post**
**1,000-m TIME**				
VO_2peak_ (mL·kg^−1^·min^−1^)	57.2 ± 6.7	58.0 ± 6.3	56.2 ± 5.8	57.7 ± 6.7
VO_2peak_ (L·min^−1^)	4.5 ± 0.8	4.4 ± 0.8	4.3 ± 0.7	4.4 ± 0.8
HR_peak_ (beat ·min^−1^)	184 ± 4	187 ± 6^**^	190 ± 8	188 ± 5
RPE (6–20)	18.5 ± 1.0	18.5 ± 1.5	19.0 ± 1.0	18.5 ± 1.0
**RUNNING VO_2max_ TEST**				
VO_2max_ (mL·kg^−1^·min^−1^)	64.1 ± 5.1	64.7 ± 5.6	63.2 ± 5.2	63.9 ± 5.0
VO_2max_ (L·min^−1^)	4.9 ± 0.8	5.0 ± 0.8	4.9 ± 0.9	4.9 ± 0.9
HR_peak_ (beat ·min^−1^)	190 ± 5	188 ± 5	190 ± 8	188 ± 8^*^
RPE (6–20)	18.5 ± 1.0	19.0 ± 1.0^*^	19.0 ± 0.5	19.0 ± 1.0

MET, Combined muscular endurance and endurance training group; ET, endurance training group.

* Significant in-group difference from pre- to post-test (P < 0.05).

** *Significant difference in pre- to post-test change between groups (P < 0.05)*.

### Physiological response during submaximal double poling

**Figure 6:**
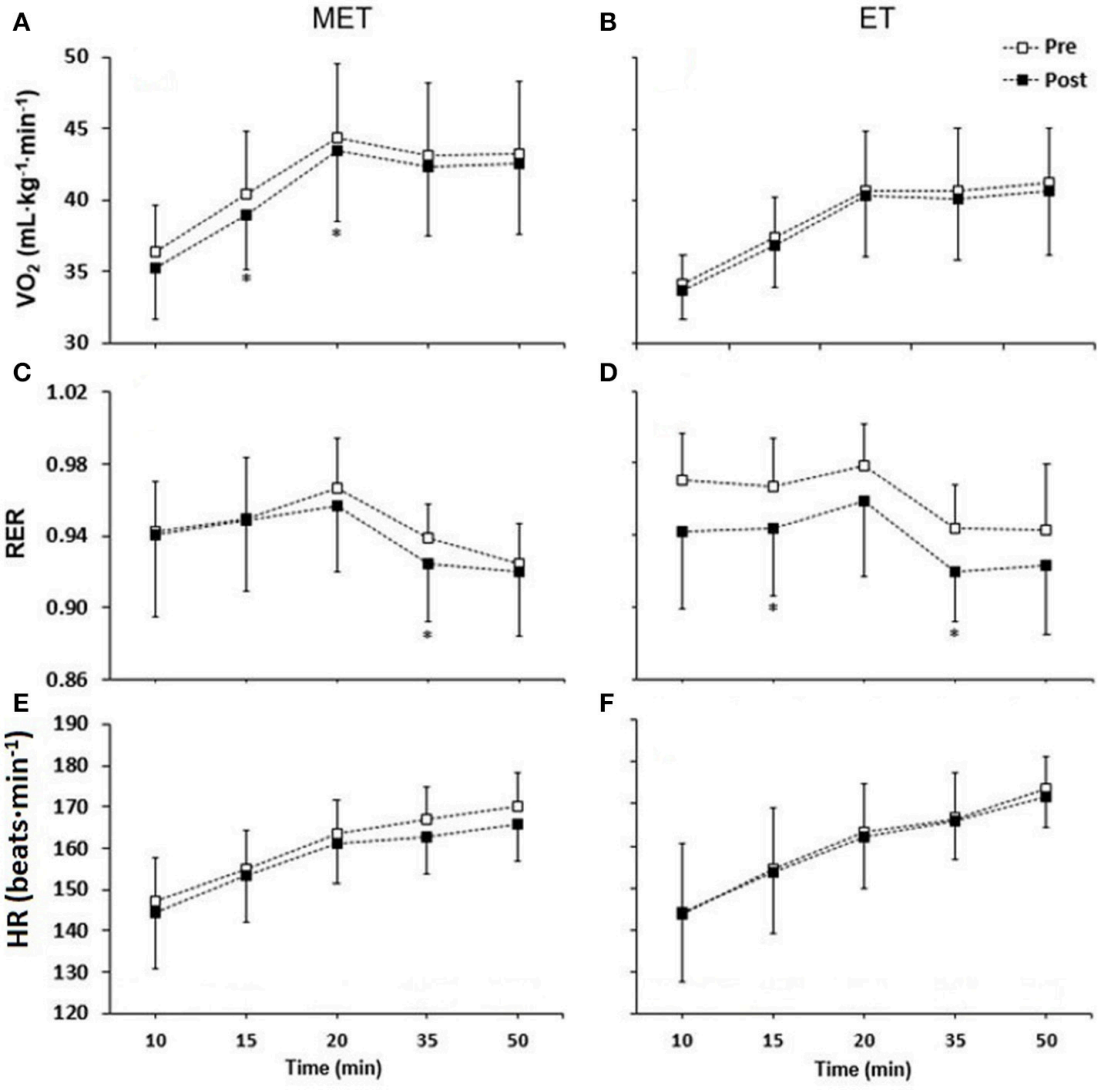
O2-cost; VO_2_ **(A,B)**, respiratory exchange ratio, RER **(C,D)** and heart rate, HR **(E,F)** during the prolonged 50-min double poling protocol before (pre-test) and after (post-test) the 6-week intervention period. MET, Combined endurance training and muscular endurance training (left), ET, endurance training (right). Data are expressed as group mean ± standard deviation. ^*^Different from pre-test (*P* < 0.05).

**Figure 7:**
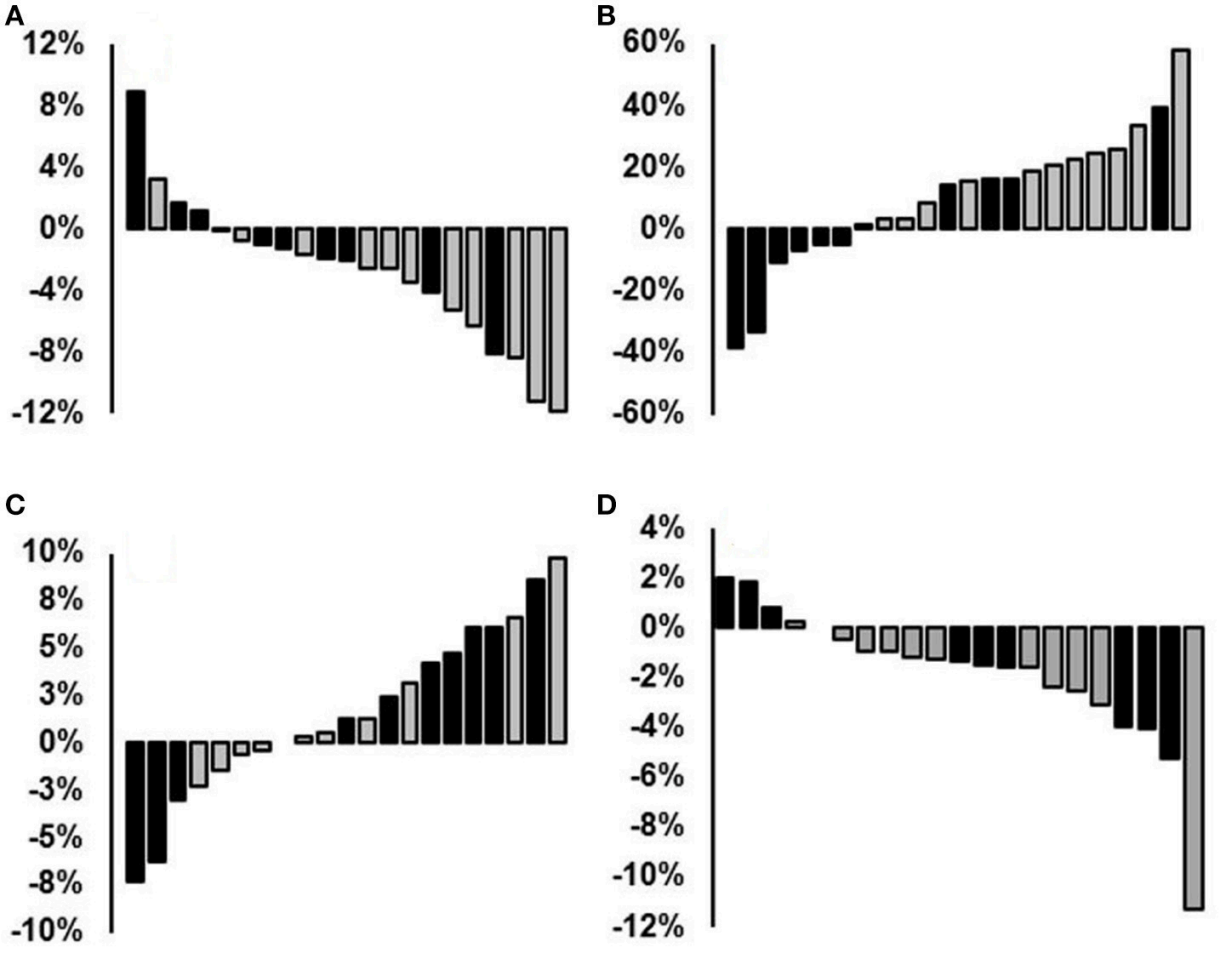
Percent individual changes from pre- to post-test: **(A)** 1,000-m time, **(B)** muscular endurance, **(C)** VO_2peak_ double poling (mL·kg^−^^1^·min^−^^1^) and **(D)** average O_2_-cost from the following measuring intervals: 8 to 10, 13 to 15, 18 to 20, 33 to 35, and 48 to 50 min. MET, Combined endurance training and muscular endurance training (gray columns); ET, endurance training (black columns).

The average O_2_-cost for all five submaximal measurements were reduced in MET from pre- to post-test, (−2 ± 2%) mainly due to reduced O_2_-cost after 15 (−3 ± 2%) and 20 min (−2 ± 1%, Figure 6A). No significant change was found in ET for mean O_2_-cost (−1 ± 1%), or at any specific time-point (Figure 6B). For both groups, individual changes in average O_2_-cost are shown in Figure 7C. However, there was no significant change between groups in mean O_2_-cost or O_2_-cost for single time measurements. There was a decrease in RER in MET after 35 min (−2 ± 1%, Figure 6C), and in ET after 15 (−2 ± 1%) and 35 min (−2 ± 1%, Figure 6D), resulting in a larger decrease in ET than in MET after 15 min (2 ± 1%). Heart rate remained unchanged from pre- to post-test for both groups (Figures 6E,F). Cycle time was not different from pre- to post-test both within and between groups.

## Discussion

This study investigated the effect of replacing parts of high-intensity interval training with upper-body muscular endurance training in well-trained XC skiers. The principal findings were: (I) Six weeks of muscular endurance training increased muscular endurance and maximal strength in a simulated DP exercise. (II) MET tended to improve 1,000-m DP performance after 50 min of submaximal DP compared to ET. (III) MET reduced the O_2_-cost during submaximal DP, but it was not significant different from ET. (IV) No changes in VO_2peak_ DP or VO_2max_ running were found in either group.

A novel finding of the present study was that upper-body muscular endurance training improved 1,000-m poling time completed immediately after 50 min of submaximal DP. In a similar study, but with a slightly different training model, Nilsson et al. (2004) found a significant improvement in mean power output during a 6-min all-out test after 6 weeks of 20-s DP interval training. Moreover, Vandbakk et al. (2017) showed that 30-s DP intervals over ~8 weeks resulted in improved time to exhaustion in the interval group, but not in the control group. Together, these studies indicate that short-term upper-body endurance training may be a promising training model and, thus, have direct applications for well-trained skiers aiming to improve their DP performance. However, whether these findings on an indoor treadmill are valid on snow needs to be examined.

The improved 1,000-m time should be a result of improved energy turnover and/or reduced O_2_-cost (Bassett and Howley, 2000). No significant changes were measured in aerobic energy turnover in either group while the anaerobic capacity was not estimated (due to methodology). However, the relative change in O_2_-cost during prolonged DP was in favor the group that had trained upper-body muscular endurance, possibly contributing to the enhanced performance due to a lower level of fatigue before the 1,000-m time. These findings are in accordance with Rønnestad et al. (2011) who showed increased 5 min all-out performance following 185 min of cycling after a heavy strength training intervention. However, mechanisms for increase in work economy, when including heavy strength training or muscular endurance training, remain unclear. Furthermore, in both the present study and in Rønnestad et al. (2011) the reduction in O_2_-cost should be taken with caution since the relative differences in O_2_-cost between groups was not significant.

The ratio VO_2peak_ DP/VO_2max_ running was ~90% at pre-test, which is similar to other studies (Rud et al., 2014; Skattebo et al., 2015). However, no changes were observed in either group, a finding consistent with most studies on heavy strength training, short-term speed training or endurance training in XC skiing (Hoff et al., 1999; Østeras et al., 2002; Nilsson et al., 2004; Rønnestad et al., 2012; Skattebo et al., 2015). Thus, reducing this “gap” has been speculated to be one of the possible training benefits of increased upper-body training in XC skiers (Sandbakk and Holmberg, 2017). However, there is very little information on which type of training is most effective in stimulating these adaptations, and future studies should examine possible mechanisms more in detail. Interestingly, both groups displayed the same VO_2max_ in running after the training intervention despite MET replaced half of the high-intensity interval sets with upper-body muscular endurance training. This may be important information regard tapering strategies where reduction in volume, while maintaining intensity and training frequency, have been proposed to induce an “optimal” strategy in the final weeks before competition (Bosquet et al., 2007).

The relative increase in muscular endurance per session (~1.8%) is larger than previous reported in comparable studies on specific upper-body training (0.7–1.1%) (Stone and Coulter, 1994; Schoenfeld et al., 2015). However, Schoenfeld et al. (2015) and Stone and Coulter (1994) included 24 and 27 training sessions respectively, compared to 12 in the current study. It is therefore possible that athletes could gain a relative large increase in muscular endurance within a few sessions. This notion is particularly interesting from a “block periodization” perspective, herein shorter training periods (1–4 weeks) are utilized to focus on improving a few selected abilities (Issurin, 2010). Moreover, even though the aim of the training was to increase muscular endurance, strength gain (1RM) per session in the current study was similar to studies in heavy strength training for XC skiers (0.6 vs. 0.5–1.2% per session; Losnegard et al., 2011; Rønnestad et al., 2012; Skattebo et al., 2015). This implies that low resistance strength training could be an alternative training method to heavy strength training, at least for short-term adaptations prior to competitions or during short block periodization.

### Methodological considerations

We counter-balanced the two groups based on 1,000-m time, VO_2max_ running, 1RM and gender at pre-test. No significant differences between groups were found in training volume in any of the training categories. However, since the subjects were well-trained and not elite athletes, the range between subjects was large in most categories, causing substantial variation in- and between groups. The absolute difference in total training volume of 132 min/week [485 min (MET) vs. 353 min (ET)] prior to the intervention period was mainly caused by a 107 min difference in weekly DP training on rollerski. This can indicate that MET had less potential for physiological adaptation than ET, which strengthens findings of the improved 1,000-m. On the other hand, MET reduced weekly DP training by 50 min (−27%) from pre-intervention compared to the intervention period, and a reduction in training volume is related to tapering and potentially improved performance (Bosquet et al., 2007; Mujika, 2010). Altogether, using recreational but well-trained skiers with large variations in training load may potentially be a limitation in the present study and should therefore be taken into consideration when interpreting the results. Another aspect is that HIT was replaced with muscular endurance, and not that training was added to their normal training, as done in most other studies. This was based on the fact that recreational athletes normally have limited time to execute training (e.g., full time work) and thereby relative low training volume compared to elite athletes. Hence, adding training in one group would lead to an increase in total volume, which could lead to a greater training stimuli it selves, and potentially enhanced performance. Finally, one strengthen of the present study is the applied perspective with direct practical application for coaches and athletes that aim on optimizing performance. However, one clear limitation of the study is the lacking methodology to analyse possible changes in intrinsic factors (such as muscle fiber types, mitochondria, capillary density and neuromuscular characteristics). Hence, since this was out of the scope of the present study, further studies should investigate the potential adaptations more in detail.

## Conclusion

Six weeks of upper-body muscular endurance training increased muscular endurance and maximal strength in a simulated DP exercise and improved DP performance following 50 min submaximal trial. Finally, replacing half of the running interval sets with upper-body muscular endurance training had no negative effects on the skiers' VO_2peak_ in DP or VO_2max_ running.

## Ethics statements

This study was carried out in accordance with the recommendations of Regional Committee for Medical and Health Research Ethics, Norwegian Research Ethics Act (2006) and Act on Medical and Health Research (2008) with written informed consent from all subjects. The study was conducted according to the Declaration of Helsinki and Norwegian law.

## Author contributions

We hereby state that the contributions from the authors are in line with author guidelines as described below. The conception or design of the work; or the acquisition, analysis, or interpretation of data for the work; JB, SNJ, BR, and TL Drafting the work or revising it critically for important intellectual content; JB, SNJ, BR, and TL Final approval of the version to be published; JB, SNJ, BR, and TL Questions related to the accuracy or integrity of any part of the work are appropriately investigated and resolved; JB, SNJ, BR, and TL.

### Conflict of interest statement

The authors declare that the research was conducted in the absence of any commercial or financial relationships that could be construed as a potential conflict of interest.
